# Functional analysis of AEBP2, a PRC2 Polycomb protein, reveals a Trithorax phenotype in embryonic development and in ESCs

**DOI:** 10.1242/dev.123935

**Published:** 2016-08-01

**Authors:** Anne Grijzenhout, Jonathan Godwin, Haruhiko Koseki, Michal Ryszard Gdula, Dorota Szumska, Joanna F. McGouran, Shoumo Bhattacharya, Benedikt M. Kessler, Neil Brockdorff, Sarah Cooper

**Affiliations:** 1 Developmental Epigenetics, Department of Biochemistry, University of Oxford, Oxford OX1 3QU, UK; 2 Laboratory for Developmental Genetics, RIKEN Center for Integrative Medical Sciences, 1-7-22 Suehiro, Tsurumi-ku, Yokohama 230-0045, Japan; 3 Department of Cardiovascular Medicine andWellcome Trust Centre for Human Genetics, University of Oxford, Oxford OX3 7BN, UK; 4 TDI Mass Spectrometry Laboratory, Target Discovery Institute, Nuffield Department of Medicine, University of Oxford, Oxford OX3 7BN, UK

**Keywords:** Chromatin, Polycomb, Trithorax, PRC2, AEBP2, Mouse

## Abstract

The Polycomb repressive complexes PRC1 and PRC2 are key mediators of heritable gene silencing in multicellular organisms. Here, we characterise AEBP2, a known PRC2 co-factor which, *in vitro*, has been shown to stimulate PRC2 activity. We show that AEBP2 localises specifically to PRC2 target loci, including the inactive X chromosome. Proteomic analysis confirms that AEBP2 associates exclusively with PRC2 complexes. However, analysis of embryos homozygous for a targeted mutation of *Aebp2* unexpectedly revealed a Trithorax phenotype, normally linked to antagonism of Polycomb function. Consistent with this, we observe elevated levels of PRC2-mediated histone H3K27 methylation at target loci in *Aebp2* mutant embryonic stem cells (ESCs). We further demonstrate that mutant ESCs assemble atypical hybrid PRC2 subcomplexes, potentially accounting for enhancement of Polycomb activity, and suggesting that AEBP2 normally plays a role in defining the mutually exclusive composition of PRC2 subcomplexes.

## Abstract (precis)

**Highlighted article:** Targeted mutation of the Polycomb protein AEBP2 in mouse provides evidence for a role for this factor in defining the composition and activity of PRC2 complexes.

## INTRODUCTION

Polycomb group proteins, which were initially identified for their role in heritable silencing of Hox gene clusters in *Drosophila melanogaster*, are highly conserved in vertebrates (Simon and Kingston, 2009). There are ∼1500-2000 Polycomb target genes in mouse embryonic stem cells (ESCs), and the majority of these encode important developmental regulators (Ku et al., 2008). Polycomb proteins confer epigenetic repression by modification of chromatin. The Trithorax group proteins act to antagonise Polycomb function and have a role in developmental gene activation (Steffen and Ringrose, 2014).

There are two major Polycomb repressive complexes, PRC1 and PRC2, which ubiquitylate histone H2A lysine 119 (H2AK119u1) and methylate histone H3 lysine 27 (H3K27me1/2/3), respectively. Six major variant PRC1 complexes, defined by the presence of different Polycomb group RING-finger subunits (Gao et al., 2012), are present in mammals and are thought to have distinct roles in targeting/maintenance of H2AK119u1 (Isono et al., 2013; Blackledge et al., 2014). The PRC2 core complex consists of the catalytic SET domain-containing subunit EZH1/2 and core subunits EED, SUZ12 and RbAp46/48 (also known as RBBP7/4). Additionally, there are non-stoichiometric accessory factors that associate with PRC2. In mammals, these include AEBP2, JARID2, PHF1 (PCL1), MTF2 (PCL2), and PHF19 (PCL3) (Shen et al., 2009; Casanova et al., 2011; Ballaré et al., 2012; Cai et al., 2013; Alekseyenko et al., 2014).

The roles of the different PRC2 accessory proteins are not well defined, although there is evidence that they can stimulate the catalytic activity of the core complex or can play a role in PRC2 recruitment (Cao and Zhang, 2004; Nekrasov et al., 2007; Casanova et al., 2011; Li et al., 2011; Brien et al., 2012; Hunkapiller et al., 2012). For example, human PCL1 (PHF1) has been shown to enhance enzymatic activity of PRC2 *in vitro*, and *PCL1* knockdown leads to reduced levels of H3K27me3 (Cao et al., 2008; Sarma et al., 2008). JARID2 depletion has no effect on global H3K27me3 levels, although both reduced and increased H3K27me3 levels have been reported to occur at Polycomb target genes in *Jarid2* knockouts or knockdowns (Peng et al., 2009; Shen et al., 2009; Landeira et al., 2010; Li et al., 2010; Pasini et al., 2010). Interestingly, a recent report has implicated a PRC2 complex containing the substoichiometric subunits AEBP2 and JARID2 in binding to nucleosomes containing the modification deposited by PRC1, H2AK119u1 (Kalb et al., 2014). Moreover, AEBP2, and to a lesser extent JARID2, were found to stimulate PRC2 activity on H2AK119u1-modified nucleosomes *in vitro*.

The substoichiometric PRC2 subunit AEBP2 has a long and short isoform, both containing a zinc-finger domain and a K/R-rich domain, which are highly conserved in mammals. These domains are also present in the C terminus of a homologous protein (Jing) in *Drosophila*. AEBP2 was first identified in a screen for factors that bind to the upstream region of the adipose *aP2* gene (also known as *Fabp4*) (He et al., 1999). Based on the proposed ability of the zinc-finger domain to bind to DNA, it has subsequently been suggested that AEBP2 could play a role in recruitment of PRC2 (Kim et al., 2009). Homozygous *Aebp2* loss of function led to early embryonic lethality but Polycomb phenotypes were not reported (Kim et al., 2011). Therefore, the role of AEBP2 in Polycomb biology remains largely undefined.

In *Drosophila*, removal of Jing leads to a variety of developmental phenotypes, including a defect in border cell migration and in differentiation of CNS midline neurons and glia. Interestingly, Culi et al. (2006) showed that *jing* mutants genetically interact with mutants in the PRC1 component Polycomb and display a similar yet milder phenotype in the wing. A recent study reported that heterozygote *Aebp2* mutant mice display an array of defects indicative of a role for AEBP2 in neural crest development (Kim et al., 2011).

Here, we report that homozygous mutant *Aebp2* mice unexpectedly display a Trithorax phenotype. Consistent with this, we observe an increase of H3K27me3 at PRC2 target genes in *Aebp2* mutant mouse (m)ESCs. Our biochemical analysis shows that AEBP2 is exclusively in the PRC2 complex and is present at the promoters of PRC2 target genes. Importantly, we demonstrate a role for AEBP2 in defining PRC2 accessory subunit composition. We suggest that perturbance of this function in *Aebp2* mutant ESCs may lead to aberrant PRC2 activity, which could account for the observed increase of H3K27me3 at PRC2 targets and the Trithorax phenotype.

## RESULTS AND DISCUSSION

### Removal of AEBP2 in mice leads to a Trithorax phenotype

**Fig. 1.**
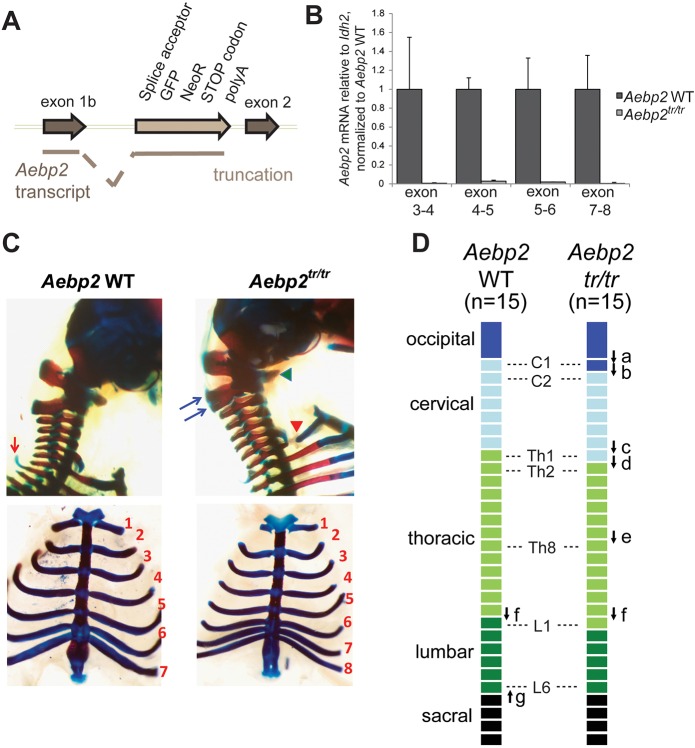
***Aebp2* truncation leads to perinatal lethality and anterior homeotic transformations.** (A) Insertion of the splice acceptor cassette in front of exon 2 leads to trapping of the transcript and a protein product that contains the first 217 amino acids (aa) of AEBP2, encoded in exon 1b, fused to green fluorescent protein (GFP) and aminoglycoside 3′ phosphotransferase (NeoR). (B) The levels of *Aebp2* mRNA transcripts containing exons downstream of the trapping cassette are severely reduced in *Aebp2^tr/tr^* mESCs compared with the parental WT *Aebp2* mESCs. Error bars indicate s.d. (C) Lateral views of the occipito-cervical region (top panels) and ventral views of the rib cage (bottom) of *Aebp2* WT (left) and *Aebp2^tr/tr^* (right) foetuses. In *Aebp2^tr/tr^* foetuses, the ventral region of the atlas (C1, indicated by a green arrowhead) is fused to that of the axis (C2). Ventral ossification centre of the atlas was laterally expanded and acquired similar features to occipital bone. The dorsal region of the axis was cranio-caudally expanded and partially bifurcated (indicated by blue arrows). The proximal region of the rib associated with the first thoracic vertebra (Th1) was not formed (indicated by a red arrowhead). The prominent dorsal process, which is associated with the Th2 in the *Aebp2* WT (indicated by a red arrow), is not formed in the *Aebp2^tr/tr^*. Bottom panel also shows association of the 8th rib to the sternum. (D) Schematic summary for homeotic transformations of the axis in the *Aebp2^tr/tr^* foetuses.

To investigate the role of AEBP2 within the PRC2 complex, we established a knockout mouse model that truncates *Aebp2* transcripts for both the long and short isoforms before exon 2 (*Aebp2^tr^*), resulting in a protein that does not contain the conserved zinc-finger and KR domains (Fig. 1A; Fig. S1A). As expected, no *Aebp2* transcripts containing downstream exons 3-8 were detected in homozygous mESCs (Fig. 1B). In crosses between *Aebp2^tr/+^* heterozygote mice, no *Aebp2^tr/tr^* homozygotes were recovered after weaning (compared with 45 *Aebp2^+/+^* and 85 *Aebp2^tr^^/+^*), demonstrating a requirement for AEBP2 in normal development. We went on to characterise embryos isolated at different developmental stages. *Aebp2^tr/tr^* homozygotes were recovered up until late gestation/early postnatal stages (8 *Aebp2^+/+^*, 18 *Aebp2^tr^/^+^* and 7 *Aebp2^tr/tr^* at E15.5; 22 *Aebp2^+/+^*, 50 *Aebp2^tr^/^+^* and 16 *Aebp2^tr/tr^* at E18.5). This finding contrasts with a previous analysis of an *Aebp2* gene trap mouse line (inserted into intron 1) in which embryonic lethality occurred before E10.5 (Kim et al., 2011). This difference may be attributable to use of a dissimilar genetic background and/or mutant allele, particularly given that the authors also observed enlarged colon and hypopigmentation in heterozygotes, which we do not observe. To investigate post-natal lethality in *Aebp2^tr/tr^* animals further, we carried out magnetic resonance imaging (MRI) and micro computed tomography (microCT) analysis at E15.5 (Fig. S1B). Although we did not observe major defects, mutant embryos (5/7) had enlarged jugular lymphatic sacs and two embryos also showed oedema, which together may indicate abnormal cardiac function.

A classical Polycomb phenotype in mice is posterior transformation of the skeleton, which is associated with misexpression of Hox cluster genes, and is seen in several PRC1 and PRC2 mutant embryos (van der Lugt et al., 1994; Suzuki et al., 2002; Li et al., 2011). Surprisingly, we observed that *Aebp2^tr/tr^* embryos exhibit the converse phenotype, anterior transformation (Fig. 1C,D; Table 1), which is normally associated with mutation of Trithorax factors that oppose Polycomb function (Ringrose and Paro, 2004). This observation is unexpected given evidence from *in vitro* assays demonstrating that AEBP2 stimulates PRC2 activity (Cao and Zhang, 2004; Kalb et al., 2014).

**Table 1.**
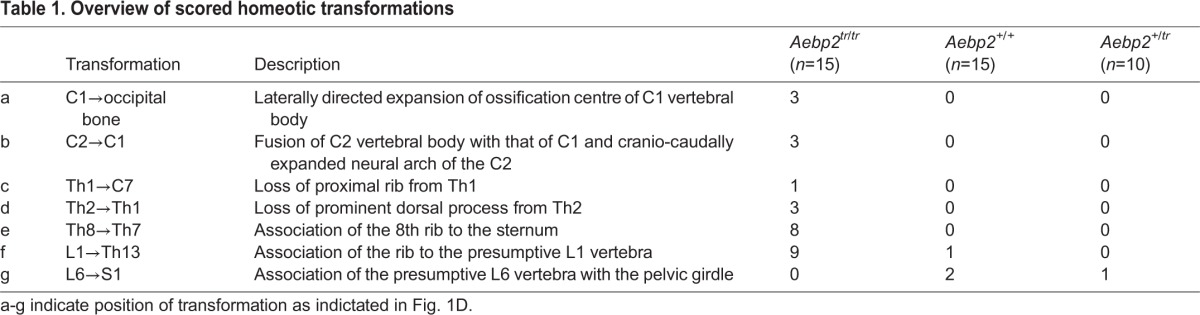
**Overview of scored homeotic transformations**

### AEBP2 is exclusively part of the PRC2.2 subcomplex

To investigate whether AEBP2 might interact with previously undefined factors that could account for the Trithorax phenotype, we analysed the AEBP2 interactome. We purified Flag-2xStrepII-tagged (FS2) AEBP2 expressed in mESCs and used tandem mass spectrometry (LC-MS/MS) to analyse interacting proteins. We identified core PRC2 and JARID2 subunits but found no Trithorax proteins or any other major interacting proteins (Table 2). In addition, gel filtration analysis demonstrated that AEBP2 co-fractionates with PRC2 core components only (Fig. S2A,B). Together, these data suggest that AEBP2 is principally a subunit of PRC2 complexes.

**Table 2.**
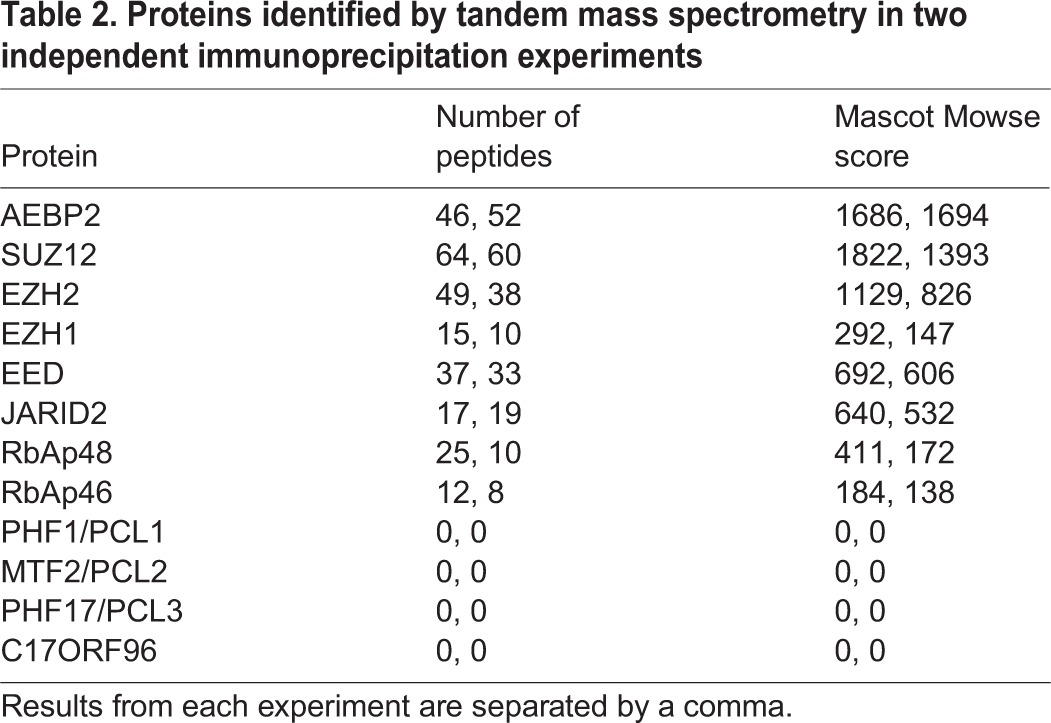
**Proteins identified by tandem mass spectrometry in two independent immunoprecipitation experiments**

**Fig. 2.**
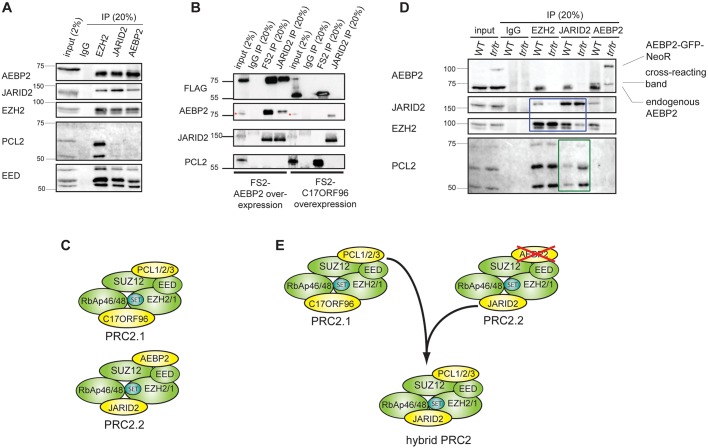
**AEBP2 is part of the PRC2.2 subcomplex and defines accessory substoichiometric subunit association.** (A) Immunoprecipitation of endogenous PRC2 subunits EZH2, JARID2 and AEBP2 in E14 mESCs. Bands of different molecular weight seen in the immunoblots of PCL2 and EED represent different isoforms of the proteins. (B) Immunoblot of co-immunoprecipitation of FS2-tagged AEBP2 (PRC2.2) and FS2-tagged C17ORF96 (PRC2.1) using either the Flag-2xStrepII (FS2) antibody, or the JARID2 antibody, illustrating that each complex associates with different PRC2 factors. The red asterisks indicate cross-reacting bands (see also Fig. S2B). (C) Schematic of PRC2.1 and PRC2.2 subcomplexes. (D) Immunoblot analysis of co-immunoprecipitation experiments to analyse the composition of PRC2 complexes. The blue box highlights the lanes that show a decreased association of EZH2 and JARID2 in *Aebp2^tr/tr^* mESCs, and the green box highlights the lanes showing an increased association of PCL2 and JARID2 upon loss of AEBP2. (E) Model of altered complex composition after AEBP2 depletion.

Notably, we did not detect PCL1/2/3 or the C17ORF96 homologue protein, which have previously been identified as PRC2 interactors (Table 2). This is consistent with reports in which purification of PCL1/2/3 and C17ORF96 did not detect AEBP2 (Casanova et al., 2011; Brien et al., 2012; Cai et al., 2013; Smits et al., 2013; Alekseyenko et al., 2014). We validated our mass spectrometry analysis by endogenous immunoprecipitation. Although EZH2 clearly immunoprecipitated PCL2 (Fig. 2A), there was no detectable PCL2 in the AEBP2 immunoprecipitate and JARID2 showed a very weak interaction with PCL2. We also used stably expressing FS2-AEBP2 and FS2-C17ORF96 homologue mESC lines to show that tagged AEBP2 did not immunoprecipitate PCL2, and that tagged C17ORF96 homologue did not immunoprecipitate AEBP2 or JARID2 (Fig. 2B). Therefore, consistent with previous studies (Alekseyenko et al., 2014), we conclude that there are two major PRC2 subcomplexes, containing PCL/C17ORF96 and JARID2/AEBP2, that we refer to as PRC2.1 and PRC2.2, respectively (Fig. 2C). Furthermore, AEBP2 is exclusively part of this PRC2.2 complex.

### AEBP2 defines PRC2 accessory subunit composition

To investigate PRC2 subunit composition in the absence of AEBP2, we analysed homozygous mutant *Aebp2* mESCs. The *Aebp2^tr/tr^* mESCs exhibited normal growth characteristics, cellular morphology and self-renewal capacity (Fig. S3A). Gel filtration analysis indicated that core PRC2 complexes are not significantly altered in *Aebp2^tr/tr^* compared with wild-type (WT) mESCs (Fig. S3B). Co-immunoprecipitation analysis, however, revealed two compositional differences in the association of accessory PRC2 factors in *Aebp2^tr/tr^* mESCs. First, association of JARID2 with PRC2 was clearly reduced (Fig. 2D, blue boxed bands). Second, we observed relatively high levels of PCL2 in JARID2-containing complexes (Fig. 2D, green boxed bands). These compositional changes suggest that AEBP2 plays a role in promoting JARID2 inclusion into PRC2.2 and that, in the absence of AEBP2, there are elevated levels of a hybrid PRC2 complex containing both PCL2 and JARID2 subunits (Fig. 2E).

### AEBP2 is enriched at PRC2 targets and the inactive X chromosome

**Fig. 3.**
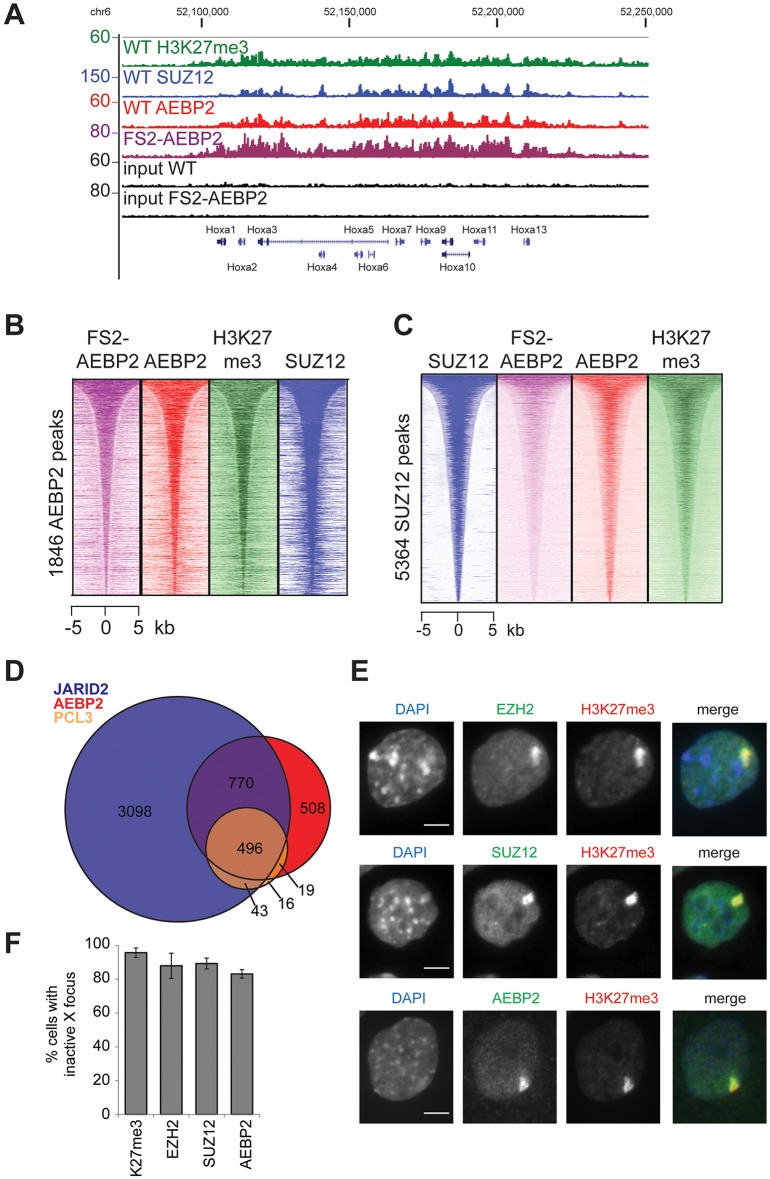
**AEBP2 is enriched at PRC2 target sites.** (A) ChIP-seq profile for SUZ12, H3K27me3, AEBP2 and input, and FS2-AEBP2 and input at the *HoxA* locus. Two repeats of ChIP-seq for the endogenous proteins were performed. Traces from one repeat are shown. (B) Heat-map analysis of AEBP2 peaks (1846), showing ChIP-seq signal for FS2-tagged AEBP2, AEBP2, H3K27me3 and SUZ12 at a 10 kb region centred over the AEBP2 peaks. (C) Heat-map analysis of SUZ12 peaks (5364), showing ChIP-seq signal for FS2-tagged AEBP2, AEBP2 and SUZ12 at a 10 kb region centred over the SUZ12 peaks. AEBP2 and FS2-AEBP2 are enriched at SUZ12 sites. (D) Overlap of peak datasets of JARID2 (Peng et al., 2009), AEBP2 and PCL3 (Brien et al., 2012). (E) Immunofluorescence images indicating overlap of H3K27me3, which marks the inactive X chromosome in trophoblast stem cells, and the PRC2 proteins EZH2, SUZ12 and AEBP2. Scale bar: 5 µm. (F) Quantification of the number of trophoblast stem cells observed with inactive X focus. A minimum of 300 cells were counted in three biological repeats. Error bars indicate s.d.

To investigate the genome-wide binding profile of the specific AEBP2-containing PRC2.2 complex, we performed chromatin immunoprecipitation sequencing (ChIP-seq) analysis in mESCs, both for epitope-tagged and endogenous AEBP2, and, as a control, SUZ12 and H3K27me3. The broad occupancy over CpG island target promoters observed for other PRC2 components is also evident for AEBP2, for example at the *HoxA* locus (Fig. 3A) and the *Gata6* promoter (Fig. S3C). Recruitment of AEBP2 to the Hox genes is consistent with the homeotic transformations observed in the *Aebp2^tr/tr^* embryos. The majority of AEBP2-bound sites are also bound by SUZ12 and H3K27me3 (Fig. 3B), suggesting that AEBP2 does not localise to sites other than PRC2 targets. Similarly, the majority of SUZ12 sites show enrichment for AEBP2 (Fig. 3C) and, consistent with this, 79.2% of published bivalent promoters (Brookes et al., 2012; Mikkelsen et al., 2007) that contain an H3K27me3 peak in our cells also contain a peak of AEBP2 (Fig. S3D). Peaks of PCL3, a component on PRC2.1, almost entirely overlap with AEBP2 and JARID2 peaks (Fig. 3D), suggesting that PRC2.2 binds to the same sites as PRC2.1.

Both PRC1 and PRC2 proteins are highly enriched on the inactive X chromosome (Xi) in cells of female mammals (Mak et al., 2002; de Napoles et al., 2004). Mouse female trophoblast stem cells contain an inactivated X chromosome that can be visualised by immunofluorescence of H3K27me3. In these cells, we showed that AEBP2, similar to EZH2 and SUZ12, is enriched on the inactive X chromosome (Fig. 3E,F). Taken together with our genome-wide ChIP-seq data, this suggests that the PRC2.2 subcomplex localises to the majority of all PRC2 sites in stem cells.

### Loss of AEBP2 leads to a small increase of H3K27me3 at PRC2 target genes

**Fig. 4.**
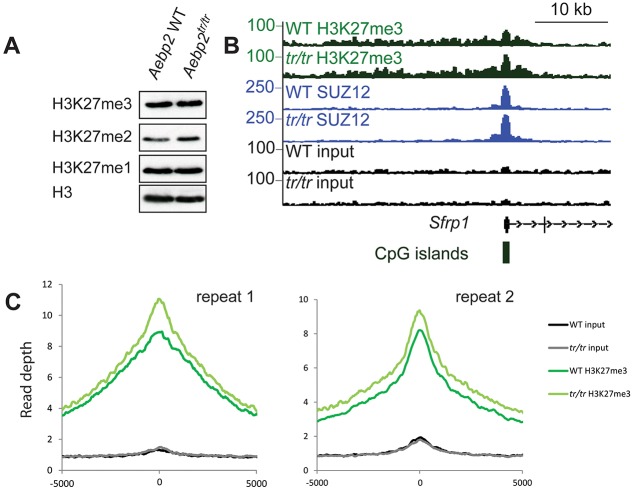
***Aebp2^tr/tr^* cells show a small increase in H3K27me3 at PRC2 target sites.** (A) Immunoblot of global H3K27me0/1/2/3 levels in *Aebp2* WT and *Aebp2^tr/tr^* mESCs. (B) ChIP-seq profile for H3K27me3, SUZ12 and input in *Aebp2* WT and *Aebp2^tr/tr^* mESCs at the *Sfrp1* gene. (C) A metaplot of H3K27me3 ChIP-seq signal at AEBP2 sites in *Aebp2* WT and *Aebp2^tr/tr^* mESCs plotted across a 10 kb window for two repeats of the ChIP-seq experiment.

Mutations in PRC2 core components result in a complete loss of H3K27me3, whereas removal of JARID2 shows no difference in global H3K27me3 levels. Similarly, we observed that *Aebp2^tr/tr^* mESCs showed no detectable variation in global levels of H3K27me1/2/3 (Fig. 4A) and no changes in the expression of PRC2, PRC1 or Trithorax-group proteins (Fig. S4A). Consistent with this observation, expression of PcG target genes in *Aebp2^tr/tr^* mESCs was largely unaffected (Fig. S4B). *Aebp2^tr/tr^* mESCs were able to differentiate into embryoid bodies following withdrawal of leukaemia inhibitory factor (Fig. S4C). This latter observation contrasts with *Jarid2* null mESCs, which fail to differentiate, and mESCs mutant for core components *Ezh2* and *Eed*, which are able to differentiate albeit with gene expression defects (Shen et al., 2008, 2009; Peng et al., 2009; Landeira et al., 2010; Leeb et al., 2010; Li et al., 2010; Pasini et al., 2010).

ChIP-seq analysis in *Aebp2^tr/tr^* mESCs did not reveal obvious differences in distribution or localisation of PRC2 (SUZ12 and H3K27me3) in *Aebp2^tr/tr^* compared with WT mESCs. Examples of the CpG island promoter of *Sfrp1* and across the *HoxA* cluster are shown in Fig. 4B and Fig. S4D. Thus, AEBP2 is not required for PRC2 recruitment to known target sites.

Interestingly, meta-analysis of all AEBP2 target sites revealed elevated levels of H3K27me3 in the mutant compared with WT mESCs. There was no consistent change in occupancy levels for the core PRC2 subunit SUZ12 (Fig. 4C; Fig. S4E), indicating that elevated H3K27me3 is linked to increased specific activity of PRC2. An increase of H3K27me3 at Polycomb loci in the *Aebp2^tr/tr^* mESCs is an effect that has been observed in Trithorax mutants and is therefore consistent with the phenotypes we see in *Aebp2^tr/tr^* embryos (Fig. 1C,D; Table 1). Given that a previous report indicated that AEBP2 could stimulate, rather than inhibit, PRC2 activity *in vitro* (Kalb et al., 2014), we speculate that the lack of AEBP2 within the PRC2 complex would be unlikely to account for an increase of H3K27me3. Instead, we hypothesise that the formation of a hybrid JARID2/PCL2-PRC2 complex formed in the absence of AEBP2 could potentially account for the Trithorax phenotype of null embryos and elevated H3K27me3 in *Aebp2^tr/tr^* mESCs. However, we cannot exclude other possibilities that may cause this effect including over-activity of PRC1 at specific sites (even though there is no global change in H2AK119u1) or a reduced turnover of PRC2.

### Conclusions

Here, we present a detailed characterisation of the PRC2 subunit AEBP2. We show that AEBP2 is exclusively present in a PRC2 subcomplex that also contains JARID2 and which localises to all Polycomb target sites in mESCs and on the inactive X in female mouse trophoblast stem cells. Despite our evidence that AEBP2 acts solely in the PRC2 complex, we observed an unexpected Trithorax phenotype in *Aebp2^tr/tr^* mice. Consistently, we also detected an increase of H3K27me3 levels at Polycomb target sites in *Aebp2^tr/tr^* mESCs. Notably, there was no loss of SUZ12 enrichment at Polycomb target sites in *Aebp2^tr/tr^* mESCs, suggesting that AEBP2 does not function in recruitment of PRC2. We do, however, find that AEBP2 orchestrates the accessory subunit composition of PRC2, as loss of AEBP2 leads to a reduction of JARID2 association and formation of a hybrid complex including PCL2. This is consistent with the central positioning of AEBP2 within the PRC2 complex as analysed by electron microscopy (Ciferri et al., 2012). Future structural studies of PRC2, including accessory subunits, will be essential to elucidate the role of AEBP2 in defining accessory subunit composition.

## MATERIALS AND METHODS

### Mice

Mice were housed in the Biomedical Sciences Building at the University of Oxford, where all procedures were approved by local ethical review committee and licensed by the Home Office under the Animals (Scientific Procedures) Act 1986. For details of transgenic line production, see supplementary Materials and Methods.

### Cell culture

Cell lines used in this study are listed in Table S1. Cell culture methods are described in supplementary Materials and Methods. Analysis of mRNA transcripts was carried out as detailed in the supplementary Materials and Methods (see Table S4 for primers).

### The *Aebp2^tr/tr^* allele and mouse skeletal preparations

The *Aebp2^tr/tr^* allele was created by targeting a vector containing an in-frame STOP cassette to mm9 genomic coordinates 140580317-140580630 (full details of targeting vector are provided in the supplementary Materials and Methods). Targeting the *Aebp2* locus was carried out using standard procedures and is detailed in the supplementary Materials and Methods. Skeletal preparations were made from embryonic day (E)18.5 mice and analysed under a stereomicroscope as described previously (Akasaka et al., 1996).

### Mass spectrometry analysis of eptitope-tagged AEBP2 protein complexes

Full-length mouse AEBP2 (496 amino acids) with an N-terminal Flag-StrepII (FS2) tag was expressed from the mammalian expression plasmid pCAG in E14 mESCs (for details, see supplementary Materials and Methods and Table S2 for primers). Nuclear cell extract was prepared and FS2-containing protein complexes immunoprecipitated in the presence of benzonase as described by Farcas et al. (2012). The analysis of immunoprecipitated digested material was performed by LC-MS/MS (full details in supplementary Materials and Methods). Full details of size exclusion chromatography are included in the supplementary Materials and Methods.

### Antibodies

Rabbit polyclonal antibodies were raised (Eurogentec) against full-length mouse AEBP2 and purified using protein A. For details, see supplementary Materials and Methods and for validation of antibody see Fig. S2. See Table S3 for details of other antibodies used.

### Chromatin immunoprecipitation

ChIP for FS2-tagged AEBP2 was performed as described (Farcas et al., 2012) except that we used rProtein A Sepharose beads (GE Healthcare) that had been blocked for 1 h at 4°C with 1 mg/ml bovine serum albumin and 1 mg/ml yeast tRNA (Sigma). ChIP for endogenous proteins was performed as described (Stock et al., 2007), except approximate concentration after sonication was measured by measuring absorbance using the Nanodrop ND-1000 (ThermoScientific) and 200 µg of chromatin was used per immunoprecipitation and after the washes DNA was purified using ChIP DNA Clean & Concentration columns (Zymo Research) and eluted in 10 µl volume. Preparation of ChIP-seq libraries and bioinformatics analysis is described in the supplementary Materials and Methods.

### MRI and microCT

Embryos were analysed by MRI and micro-CT. E15.5 embryos, generated by timed mating of *Aebp2* heterozygous mice, were dissected into warm HANKS BSS without calcium/magnesium (Sigma, H4641) with 5 mM EDTA, warmed to 37°C, for culling by exsanguination in ice-cold PBS. Embryos were fixed for 3 days in 4% paraformaldehyde with 2 mM Gd-DTPA (gadolinium) as a contrast agent and MRI was performed as previously reported (Schneider et al., 2004). For higher resolution images, following MRI scan, selected embryos were scanned by microCT. Prior to microCT, embryos were incubated in 100% Lugol's solution for 4 days (to achieve soft tissue contrast) and embedded in a tube with 1% agarose. Datasets generated by both scanning techniques were analysed using Amira 5.3.3 software (FEI Visualization Sciences Group, Merignac, France).
